# Improving storytelling and vocabulary in secondary school students with language disorder: a randomized controlled trial

**DOI:** 10.1111/1460-6984.12471

**Published:** 2019-03-29

**Authors:** Victoria L. Joffe, Lorna Rixon, Charles Hulme

**Affiliations:** ^1^ School of Health Sciences, City University of London London UK; ^2^ Department of Education University of Oxford Oxford UK

**Keywords:** language disorder, narrative intervention, vocabulary intervention, secondary school, adolescence, teaching assistants

## Abstract

**Background:**

Although language and communication difficulties are common in secondary school students, there has been limited research into the efficacy of interventions for adolescents with language and communication difficulties.

**Aims:**

To investigate the efficacy of teaching assistant (TA)‐delivered narrative and vocabulary interventions to mainstream secondary school‐aged students with language disorder.

**Methods & Procedures:**

A randomized controlled trial (RCT) of a language and communication intervention was used to evaluate the efficacy of vocabulary and narrative interventions to improve the vocabulary and narrative performance of adolescents (mean age = 12.8 years) with language disorder. The language and communication programmes (narrative, vocabulary and combined narrative and vocabulary) were delivered by TAs in the classroom, three times per week, for 45–60 min each, over 6 weeks, totalling 18 sessions. Standardized and intervention‐specific measures were used as outcomes.

**Outcomes & Results:**

Twenty‐one schools with 358 eligible participants were recruited. The three intervention groups showed significant improvements (*d* = .296) on a narrative latent variable defined by a standardized narrative assessment (the Expression, Reception and Recall of Narrative Instrument—ERRNI), but there were no significant improvements on an overall vocabulary latent variable compared with the waiting control group. Differential effects were found on some non‐standardized intervention‐specific measures with the narrative group making significantly more progress on narrative tasks compared with the waiting control group, the vocabulary group showing the same pattern on specific vocabulary tasks, and the combined narrative and vocabulary group making significantly more progress on some of the intervention‐specific narrative, and all the intervention‐specific vocabulary outcomes compared with the waiting control group.

**Conclusions & Implications:**

It is possible to improve narrative but not vocabulary skills, as assessed by standardized measures, in secondary school students with a relatively brief group TA‐delivered intervention. There were differential effects for both narrative and vocabulary with intervention‐specific measures. Future work is required to explore whether more intensive and longer lasting interventions would be more effective and to identify which students in this age group are most likely to benefit from such interventions.

**What this paper adds**

**What is already known on the subject**

Language and communication difficulties persist into adolescence, and although there is some evidence for effective interventions in pre‐ and primary school‐aged children, there is limited evidence of effective language and communication interventions in secondary school‐aged students.

**What this paper adds to existing knowledge**

The language and communication programmes developed here are novel interventions for adolescents, and this is one of the first large randomized control trials of its kind in secondary school students with language disorder. The findings show that adolescents with language difficulties can improve their narrative skills as a result of small‐group intervention, delivered by trained TAs. Improvements were found on narrative and vocabulary skills with intervention‐specific assessments.

**What are the potential or actual clinical implications of this work?**

The results justify this choice of service delivery model as an option for adolescents with language disorder in mainstream secondary schools. The study shows that it is possible to train TAs to deliver language interventions in secondary schools which are effective and relatively low cost.

## Introduction

Although basic language and communication skills develop in the preschool years, there is increasing recognition that language and cognition continue to develop throughout adolescence and into adulthood (Blakemore and Choudhury 2006, Blakemore 2008, Nippold 2017). Language development in adolescence requires an understanding and use of increasingly complex vocabulary, figurative language (such as idioms and double meanings) and more complex sentence structures. Individuals are able to tell stories with increasing sophistication, show appreciation of emotion and motivation of characters, and draw upon advanced social skills such as persuasion and negotiation (Nippold 2017). Narrative and vocabulary skills are crucial building blocks for later language development, and have been shown to be strong longitudinal predictors of literacy and educational attainment (Croll 1995, Fazio *et al*. 1993, Lervag *et al*. 2018, Nation and Snowling 2004, Spencer *et al*. 2017a, Stothard *et al*. 1998). Students with language disorder often experience difficulties with these skills (Conti‐Ramsden *et al*. 2013, Rice and Hoffman 2015, Wetherell *et al*. 2007), and interventions focusing on broader language skills, including narrative, vocabulary and inferences, have been recommended as a means to improving literacy (Lervag *et al*. 2018). The current study builds on earlier studies with younger age groups that have shown that direct teaching of vocabulary knowledge and narrative language skills can be effective for children with weak oral language skills (Fricke *et al*. 2017, Clarke *et al*. 2010). In line with the simple view of reading (Gough and Tunmer 1986) these interventions have been shown to lead to improvements in reading comprehension as well as oral language comprehension ability.

Early language difficulties are pervasive, can persist into adolescence and adulthood, and affect academic performance, and social and emotional functioning (Conti‐Ramsden and Botting 2008, Conti‐Ramsden *et al*. 2013, Johnson *et al*. 2010). Prevalence rates for language disorder have recently been reported to be 9.92% (Norbury *et al*. 2016), with some studies reporting a higher prevalence in adolescence (e.g., McLeod and McKinnon 2007).

Adolescents with language disorder have more difficulties with peers, and emotional and behavioural problems, and report having more mental health difficulties (Conti‐Ramsden *et al*. 2013).

While interventions for language and communication difficulties have been found to have moderate to large effect sizes in primary school children, there is limited research into the effectiveness of interventions in secondary school students (Cirrin and Gillam 2008). Given the continuing development of language and communication in adolescence, the window of opportunity for improving cognitive function is much wider than once thought (Knoll *et al*. 2016).

Research investigating the effectiveness of interventions to enhance language and communication in adolescents with language and communication difficulties is emerging, particularly in the area of vocabulary (see, for example, a recent systematic review on vocabulary interventions for adolescents with language disorder by Lowe *et al*. 2018) across a range of service delivery models: specialist, targeted and universal (Gascoigne 2006). The studies cited in this systematic review were diverse and reflected interventions that focused primarily on semantics, phonology and a combined phonological–semantic approach, which links the sound and meaning of the word. Whilst the strongest evidence for the effectiveness of vocabulary intervention came from studies using a phonological–semantic approach, in individual, small group and whole‐class settings the results were, on the whole, mixed and should be viewed at this stage as preliminary (Lowe *et al*. 2018).

### Vocabulary interventions

A number of studies have shown the effectiveness of vocabulary intervention programmes, delivered by teaching staff in schools, with secondary school second‐language learners with poor vocabulary (e.g., Lesaux *et al*. 2010, 2014, Snow *et al*. 2009). More recently, a number of studies have evaluated the effectiveness of vocabulary interventions for secondary school students with language disorder (in individual, group and whole‐class settings), with mixed results. A few studies have shown one‐to‐one specialist speech and language therapy, in a specialist school for adolescents with severe language disorder, to be effective in improving word‐finding difficulties (Ebbels *et al*. 2012), vocabulary knowledge (Wright *et al*. 2018) and a broader range of specific expressive and receptive language targets (Ebbels *et al*. 2017). Mixed results were found by Spencer *et al*. (2017b), who explored the effectiveness of a vocabulary intervention in a small group model within mainstream secondary schools. The intervention group made no significant progress on a bespoke word‐learning task; however, the delayed control group, which received the same intervention at a later time, showed significant progress following their intervention. Universal models of intervention have also been shown to be effective in improving language skills of adolescents with language and communication difficulties. In a randomized controlled trial (RCT) in two schools (Starling *et al*. 2012), teachers were taught a range of oral and written language modification techniques to support adolescents with language disorder. Starling *et al*. found that the incorporation of these techniques by teachers in the classroom improved written expression and listening comprehension relative to the control group, with no changes on a standardized measure of oral expression or reading comprehension. In another study using the same universal model of service delivery as Starling *et al*., and one of the very few RCTs of vocabulary interventions, Murphy *et al*. (2017) explored the effectiveness of a whole‐class vocabulary intervention in mainstream secondary schools in areas of social disadvantage, employing an adapted shortened version of the vocabulary intervention programme (Joffe 2011b) used in the current study. A total of 203 eleven to thirteen year olds (128 in the experimental group; 75 waiting controls), with a below‐normative mean receptive vocabulary score of 83.72 on the British Picture Vocabulary Scale, 3rd edn (BPVS‐3; Dunn *et al*. 2009), received vocabulary intervention delivered by teachers in the classroom over twelve 40‐min sessions. Results were mixed, with both the experimental and waiting control group making significant progress on the Clinical Evaluation of Language Fundamentals, 4th edn (CELF‐4; Semel *et al*. 2006) and BPVS‐3. However, the waiting controls made further significant progress on the CELF‐4 and BPVS‐3 after receiving the intervention. Another similar RCT, exploring the effectiveness of the universal model, but focusing on socially disadvantaged secondary school students, also reported improvements over time in the treated group compared with controls (waiting‐list schools) on some measures of receptive and expressive vocabulary after receiving a vocabulary intervention, delivered by teachers within a mainstream whole‐class context, twice a week for 12 weeks (24 sessions) (McNamara 2014). More recently, Lowe and Joffe (2017), again using the universal service delivery model, reported some improvement in the knowledge of targeted science vocabulary, in a small pilot study, with 15 adolescents with language disorder when taught by teachers in a mainstream class using a phonological–semantic approach. Following on from this pilot study, in a larger RCT, Lowe *et al*. (2019) found significant improvements in science vocabulary in 78 adolescents with language disorder, which had been taught by teachers in mainstream classes using a phonological–semantic approach. Results from these studies in the adolescent population, across different settings, are encouraging, but are nonetheless somewhat mixed and therefore require further evidence.

### Narrative interventions

Whilst narratives have been employed effectively in interventions with pre‐ and primary school‐aged children with language disorder (e.g., Davies *et al*. 2004, Gillam *et al*. 2018, Gillam and Gillam 2016, Swanson *et al*. 2005), there are limited narrative intervention studies with adolescents. In a systematic review of narrative‐based language interventions with children with language impairment conducted by Petersen (2011), only one of nine studies included older children with this study focusing on expressing oral and written narratives (Gillam *et al*. 1995). Despite positive results, there were a limited number of participants (eight 9–12 year olds) and little experimental control.

Further mixed evidence for the effectiveness of narrative intervention in secondary school students comes from small‐scale studies by Stringer (2006) and Joffe (2006), both using a small group setting, with a focus on the comprehension and expression of oral narratives. Stringer (2006) explored the benefits of a group narrative and social skills programme to 12 secondary school‐aged children with emotional and behavioural difficulties (mean age = 12.4 years) with improvements reported on some sentence‐level language tests, but not on single‐word measures or on an oral story comprehension task. Joffe (2006) conducted an RCT with 54 adolescents (mean age = 12.8 years) with language disorder, exploring the effectiveness of a narrative and vocabulary intervention, with both groups making significant progress on receptive vocabulary, sentence recall and inferential understanding, but no differential effects reported between the two groups. Whilst the evidence emerging for the effectiveness of narrative intervention, in enhancing oral storytelling, for this older age group appears positive, participant numbers are small and the results are mixed. Therefore, similarly to vocabulary intervention, further investigation is needed to increase the evidence base for narrative.

### Using teaching assistants to support language and communication

Students with language and communication difficulties are frequently supported in the classroom by teaching assistants (TAs) (Armstrong 2008, Groom 2006). In the UK, the education system employs TAs (sometimes referred to as learning support assistants) to support teachers in the classroom, and many of them have some responsibility for pupils with additional needs. Internationally, TAs can be referred to as a teacher's aide and an educational or classroom assistant or support worker, with all of them having a similar function. Increasingly, intervention research has attempted to replicate this delivery model by exploring the effectiveness of interventions delivered by teaching support staff (e.g., Burgoyne *et al*. 2012, Clarke *et al*. 2010, 2017, Davies *et al*. 2004, Fricke *et al*. 2013, 2017). Considering the limited availability of speech and language therapy and specialist support in secondary schools (Bercow 2008, 2018), and the ecological validity of using existing school staff as agents of intervention (Alborz *et al*. 2009), the present study adopts this model by training TAs to deliver language interventions. To date, there has been no large‐scale RCT investigating the effectiveness of narrative and vocabulary intervention for adolescents with language disorder, delivered by TAs, in small groups in mainstream secondary schools.

The present study examines whether a narrative, vocabulary or combined narrative and vocabulary intervention delivered by TAs to secondary school‐aged students with language disorder improves narrative and vocabulary skills. We hypothesized that there would be differential improvements in the intervention groups on vocabulary and narrative outcomes compared with the waiting control group, with the vocabulary intervention group improving in vocabulary and the narrative intervention group in narrative. This would provide evidence for the importance of providing intervention‐specific treatments to older children with language disorder in mainstream settings, and for the role of TAs in delivering them.

## Methods

### Design

We conducted an RCT in two outer London boroughs in the UK, with narrative and vocabulary programmes delivered by TAs, three times a week over 6 weeks. There were three intervention groups and one waiting control group: narrative, vocabulary and combined narrative and vocabulary. Pre‐intervention assessments were conducted over 4 months and post‐intervention assessments over 3 months, following intervention. Ethical approval was obtained from the Senate Research Ethics Committee at City, University of London.

### Participants

#### Selection of participants

Participants were students in their first year of secondary school (year 7) and were recruited from 21 mainstream secondary schools.

There were two stages to recruitment. First, teachers were asked to identify Year 7 secondary school students with low‐ or below‐average scores on their Year 6 English National Standard Assessment Test (SAT). Teachers could also refer students who were underperforming academically in the classroom. The second stage of recruitment included students who met the above criteria and gave informed consent. These students were assessed on a range of language measures: British Picture Vocabulary Scale, 2nd edn (BPVS‐2; Dunn *et al*. 1997); Formulated Sentences (FS) and Recalling Sentences (RS) subtests of the CELF‐4 (Semel *et al*. 2006); Receptive Vocabulary (RV) and Expressive vocabulary (EV) subtests of the Test of Word Knowledge (TOWK; Wiig and Secord 1992); and the Multiple Contexts (MC) and Figurative Usage (FU) subtests of the TOWK. Students who scored ≤ 1 SD below the mean on two or more of the language tests/subtests, or ≤ 1.5 SD from the mean on any one language test/subtest, were recruited at this second stage.

### Description of sample

A total of 358 Year 7 students (mean = 12;8 [years;months], SD = 3 months) from 21 mainstream secondary schools across two outer London boroughs met the criteria. Two schools declined to participate as they did not feel they had students with language disorder. There were 226 males and 132 females. Of the group, 1% scored above average, 7% scored average, 34% were low average and the remaining 54% were below average in Year 6 on their English SAT (data were unavailable for the remaining 5% of the group). Students scoring in the average range were referred by teachers because of their underachievement in the classroom. Parents completed self‐report questionnaires which included information on maternal education, which was used as a proxy of socioeconomic status. Maternal education for the majority of the group (54%) was ‘school or college‐level’ qualifications. Thirteen per cent had mothers with ‘no further’ qualifications, and 11% had mothers with ‘university‐level’ qualifications. The performance IQ of the group was below average (mean = 84.4; SD = 14.2). The language and non‐verbal performances of the participants are listed in table 1.

**Table 1:** Verbal and non‐verbal abilities of participants

Measure	Mean	Standard deviation (SD)	Range	*N*
BPVS‐2^a^	85.1	12.3	44–144	352
CELF‐4 Formulated Sentences^b^	6.0	3.0	1–14	353
CELF‐4 Recalling Sentences^b^	6.3	2.8	1–15	353
TOWK Receptive Vocabulary^b^	7.5	2.2	3–17	357
TOWK Expressive Vocabulary^b^	5.7	1.7	3–13	357
TOWK Multiple Contexts^b^	6.1	2.1	3–12	357
TOWK Figurative Usage^b^	6.4	1.9	3–12	357
Non‐verbal WISC‐III Performance IQ^a^	84.4	14.2	53–133	355

Note: BPVS‐2 = British Picture Vocabulary Scale, 2nd edn (Dunn *et al*. 1997); CELF‐4 = Clinical Evaluation of Language Fundamentals—Fourth Edition (Semel *et al*. 2006); TOWK = Test of Word Knowledge (Wiig and Secord 1992); WISC‐III = The Wechsler Intelligence Scale for Children—Third Edition (Wechsler 1991).

a Mean = 100, SD = 15.

b Mean = 10, SD = 3.

### Description of the TAs who delivered the intervention

One TA was identified by the special educational needs coordinator from each school. The only selection criterion for the TAs was that they were employed as a TA at the school for the duration of the study. Their experience working as a TA in school ranged from 8 months to 18 years (mean = 5 years, SD = 4 years). TAs also varied in their level of education, ranging from National Vocational (work‐based) Qualifications (NVQ) to postgraduate training.

### Measures

Two types of measures were used: standardized and intervention‐specific assessments. The research assistants administered and scored the tests. Training was provided by the first author to all testers on the administration and scoring of both standardized and non‐standardized measures. For all standardized tests administration and scoring followed the manual guidelines. Intervention‐specific measures were modelled on existing standardized tests and from the literature to increase the content validity. Uniform scoring instructions were used and interrater reliability was conducted by independent researchers blind to the other raters’ scores for the intervention‐specific measures.

### Cognitive abilities

#### Wechsler Intelligence Scale of Children—Third Edition (WISC‐III)

In order to obtain a reliable estimate of performance IQ, four of the five core non‐verbal subtests of the Wechsler Intelligence Scale for Children—Third Edition (WISC‐III; Wechsler 1991) were used: Block Design, Picture Completion, Picture Arrangement and Coding.

### General language abilities

#### Clinical Evaluation of Language Fundamentals—Fourth Edition (CELF‐4)

Two subtests of the CELF‐4 were used: FS and RS. In the FS subtest, participants were asked to provide a sentence using a target word. In the RS subtest, students were required to repeat verbatim sentences of increasing length and complexity that were read aloud to them. Normative data were collected in the UK, with a mean = 10 and SD = 3 (Semel *et al*. 2006).

### Narrative outcomes

#### Expression, Reception and Recall of Narrative Instrument (ERRNI)

The ERRNI comprises a sequenced story of 15 coloured pictures that can be used to tell a story. The test assesses the ability of participants to relate, comprehend and remember a story after a delay. To reduce practice effects, the test is available in two parallel forms: the ‘fish story’ and the ‘beach story’. After retelling the story, nine comprehension questions are then given with the pictures in view. The stories are then transcribed and analyzed for narrative skills pertaining to the amount of information content relevant to the story, for both initial telling and recall. Detailed guidance on transcription and analysis provided in the manual were followed and scoring followed manual instructions. UK normative scores are provided, with mean = 100 and SD = 15. The reliability for initial storytelling and recall are .86 and .90 for the fish story and .85 and .90 for the beach story respectively (Bishop 2004).

#### Story generation task

Participants were asked to tell a story from a single picture, a sequence of pictures and six objects (mobile phone, handcuffs, car, camera, horse and feather) of which they were asked to include at least three of them in their story. Responses were recorded and transcribed verbatim. Standardized scoring instructions were devised to assess the quality of narrative skills, adapted from Stein and Glenn's (1979) story grammar framework. Components included organization of the narrative, sequencing, character descriptions, reference to time and place, discrete episodes of events, reactions and resolutions, emotional or cognitive responses of the characters, presence of a climax, integration of story elements, use of dialogue, onomatopoeia and sound effects, appropriate use of anaphoric referencing and idioms. Scores ranged from 0 to 75 for the picture and sequence stories, and from 0 to 78 for the object story task, with a bonus of three points for inclusion of up to three of the objects. Scores from the three tasks: picture, sequence of pictures and objects, were combined into one variable, called the story generation task. Cronbach's alpha were .88 and .91 for pre‐ and post‐assessment intervention points, indicating excellent internal reliability respectively.

### Narrative checklist

A checklist of explicit narrative knowledge was assessed using a 10‐item task exploring the following: understanding of what a story is, the ability to cite familiar stories, the identification of types of story genres, story components, story structure, linguistic devices to make stories more exciting, understanding of story climax, and components of active story listening. Scores ranged from 0 to 78. Cronbach's alpha were .93 and .98, for pre‐ and post‐intervention assessment points, indicating excellent internal reliability. This checklist is available from Joffe (2011a).

### Vocabulary outcomes

#### British Picture Vocabulary Scale, 2nd edn (BPVS‐2)

The BPVS‐2 assesses single‐word receptive vocabulary. The participants select a picture from a choice of four that best represents the meaning of the word given by the tester. The test is standardized on a UK sample, with mean = 100 and SD = 15. The reliability for Year 7 (11–12 year olds) is 0.89 (Dunn *et al*. 1997).

#### Test of Word Knowledge (TOWK)

Four subtests of the TOWK were used: single‐word receptive and expressive vocabulary, comprehension of words in multiple contexts and figurative language. RV was assessed using a picture selection task where participants were required to point to the picture, out of a selection of four, that matched the word given. For expressive vocabulary, students provided names for pictures provided, representing nouns and verbs. In the third subtest, words with multiple meanings, for example, ‘letter’, are given to the student who is asked to provide as many meanings as possible for that word. Finally, the figurative language subtest required the participant to choose, from a choice of two, the description that best matched a figurative expression. Normative scores were collected in the United States, with mean = 10 and SD = 3. Stimuli were presented orally to accommodate any reading difficulties. The reliabilities for 12 year olds are .88, .85, .91 and .92 for the RV, EV, MC and FU subtests respectively (Wiig and Secord 1992).

#### Vocabulary definitions task

Participants were required to provide definitions for 13 words associated with the themes covered in the vocabulary intervention, for example, ‘employer’ under the theme of careers. Scores of 2 and 1 were awarded for fully and partially correct responses respectively. Detailed scoring guidelines were provided with the criteria needed for a score of 2, for a complete and accurate definition, or 1, for an incomplete definition. A score of 1 was also given if the participant used the word correctly in a sentence, but did not provide a definition. For example, when defining the word ‘larynx’, the response, ‘part of the throat containing the vocal folds’ or ‘voice box’, scored 2. The definition ‘throat’ or ‘Adam's apple’ was given a score of 1. Scores ranged from 0 to a maximum of 26 correct. Cronbach's alpha were .94 and .81, for pre‐ and post‐intervention assessment points, indicating excellent and good internal reliability respectively.

#### Vocabulary idiom awareness

Participants were presented with 20 idioms that formed part of a wider set of idioms covered in the intervention, and were asked to explain what they mean. Idioms selected for intervention were chosen after reviewing some idiom dictionaries and discussions with speech and language therapists, and teaching staff from the participating schools, as well as the study advisory group. Idioms that were most appropriate for age and culture, that could be easily pictorially represented and were associated with the intervention themes, were included. A subgroup were used for the assessment. Total scores ranged from 0 to 20, with literal responses scoring 0. For example, ‘to be full of beans’ would be scored 1 for a response ‘lively or high in spirits’, and 0 for a literal response: ‘eaten too many beans’. Cronbach's alpha were .81 and .84, for pre‐ and post‐intervention testing points indicating good internal reliability, respectively.

#### Expressive vocabulary task

Participants were asked to name 15 pictures depicting vocabulary related to themes covered in the vocabulary intervention, for example, ‘galaxy’ for the theme of ‘space’. Scores ranged from 0 to 15. A score of 1 was given for a correct response and a score of 0 for an incorrect or no response. The interrater reliability for this measure was 100%.

#### Receptive vocabulary task

Participants were required to select one picture out of a choice of four that best matched the word given by the tester. There were 40 stimuli pertaining to themes covered in the intervention. For each item, there was a target, a semantic, phonological and unrelated distractor. Scores ranged from 0 to 40. A score of 1 was given for the correct selection and a score of 0 was awarded for the other three distractor items. The interrater reliability was 100%.

### Description of intervention

We compared a narrative and a vocabulary intervention programme for secondary school students with language and communication difficulties with a programme that combined both narrative and vocabulary intervention. The programmes were manualized and delivered by TAs with ongoing support from the research team (Joffe, 2011a, 2011b). The interventions incorporated themes closely associated with the curriculum. The narrative programme focused on the understanding and telling of stories, using a story structure, adapted from Stein and Glenn (1979), to support story generation. Students were introduced to different types of stories (fictional, non‐fictional, scripts) and narrative genres (see Joffe 2011a for further details). The vocabulary programme focused on developing key concepts and vocabulary items relevant to the educational curriculum (e.g., nutrition) and age appropriate (e.g., careers). A variety of tasks including word associations, categorization, mind‐mapping and word‐building were used to reinforce word learning. The intervention manual consisted of target vocabulary that TAs could draw upon to explore the themes of the session. TAs were encouraged to select an appropriate number of words commensurate to the pace of learning and interest of students within their group. A range of quizzes and games were provided in the manual to evaluate the learning of the target words covered in each session. Word etymology was used to facilitate independent word learning. The intervention explored different categories of words, their synonyms, antonyms, multiple meanings, definitions as well as idiomatic and figurative language (Joffe 2011b). The combined narrative and vocabulary intervention programme incorporated all key content of both the narrative and vocabulary programme, but with reduced exercises in order to practise for each individual component.

The programmes were delivered by TAs working with small groups of two to six pupils. Each session was 45–60 min and given three times per week over 6 weeks, totalling 18 sessions. The intervention was tailored to individuals using ‘step‐up’ and ‘step‐down’ activities depending on the required level of difficulty.

The TAs were trained initially for 4 days by the first author, with training covering an introduction to language and language disorder, knowledge of narrative and vocabulary skills, and strategies to support children with language disorder. Another half‐day training session was provided midway through delivery to reinforce strategies and answer questions. All 21 TAs at each school delivered each of the three experimental conditions (narrative, vocabulary and the combined narrative and vocabulary interventions).

To monitor and enhance intervention delivery fidelity, the research team kept in contact with the TAs weekly, observed them delivering the interventions on approximately three occasions and gave them written feedback. The TAs kept session plans and notes for each session and these were reviewed by the research team.

### Procedure

Schools identified the TAs; and parents, head teachers and TAs provided written consent before pupils participating in the study.

Assessments were conducted by the research team, who were blinded to group allocation. Those who assessed and enhanced fidelity of intervention, by monitoring and giving ongoing feedback, were not involved in assessments at those schools. Students were randomized to the four groups within each school: (1) vocabulary intervention, (2) narrative intervention, (3) combined narrative and vocabulary intervention, and (4) the delayed waiting control group. Each TA delivered all three interventions within their school. Assessments were conducted pre‐ and post‐intervention.

All standardized assessments were administered and scored according to manual guidelines. For non‐standardized assessments, training was provided to all testers, and standardized scoring instructions were used. A total of 10% of participant responses at each assessment point were independently scored by a second coder, blind to group allocation. Standardized scores were used to describe the sample (table 1), but raw scores are used in analyses.

## Results

**Figure 1:**
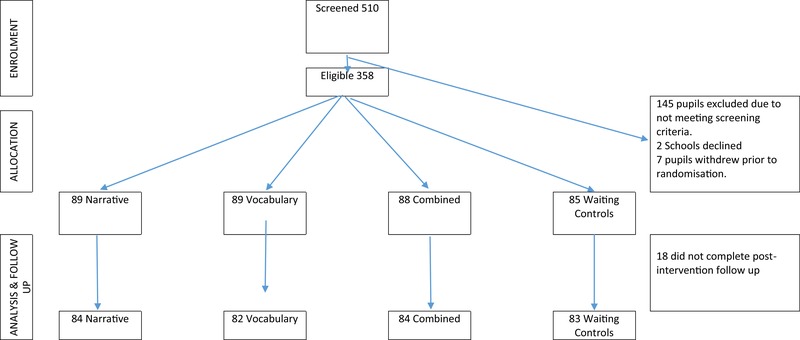
CONSORT diagram: randomized control trial of the language and communication intervention study. [Color figure can be viewed at wileyonlinelibrary.com]

Figure 1 shows the number of participants randomized to the four intervention conditions in each of the 21 schools. The rate of attrition from pre‐ to post‐intervention was 5% and Little's MCAR (Missing Completely at Random) test confirmed that missing data for all the language measures analyzed here could be considered to be missing completely at random (*χ*² = 185.79; d.f. = 177; *p* = .31). A power analysis shows that with 85 children in the waiting control group and 89 children in each of the intervention groups, using analysis of covariance (ANCOVA), and assuming a pre‐test–post‐test correlation of .6, the current study has 80% power (*p* = .05; two‐tailed) to detect a difference between groups equivalent to Cohen's *d* = .34 (a small effect).

Not all participants received the full 18 sessions of the intervention. TAs delivered 18 sessions, of which on average 15–16 sessions were attended by students in the different intervention groups. Participants in the narrative, vocabulary and combined narrative and vocabulary groups attended 15.7 (SD = 2.6), 15.2 (SD = 3.3) and 16.1 (SD = 2.2) sessions respectively. There were no significant differences in dose received between the three groups (*F*(2;221) = 1.85, *p* = 0.160).

Descriptive statistics at pre‐ and post‐test for the narrative, vocabulary, combined narrative and vocabulary and waiting control groups for standardized measures are shown in table 2, and for intervention‐specific measures in table 3.

**Table 2:** Descriptive statistics by intervention group (raw scores) for standardized assessments of narrative and vocabulary

	Control		Vocabulary			Narrative			Combined		
	Pre	Post	Pre	Post	*d* (compared with control)	Pre	Post	*d* (compared with control)	Pre	Post	*d* (compared with control)
ERRNI Initial Story Telling	25.19, SD = 5.59	24.99 SD = 6.30	25.09 SD = 5.47	26.44 SD = 6.01	0.26*	24.65 SD = 4.35	26.75 SD = 6.69	0.40*	24.42 SD = 5.69	26.17 SD = 5.37	0.34*
ERRNI Recall	22.60, SD = 7.30	21.75 SD = 6.51	24.36 SD = 8.34	23.70 SD = 5.74	0.03*	23.55 SD = 5.38	23.11 SD = 7.06	0.06*	23.10 SD = 6.36	23.48 SD = 5.60	0.19*
BPVS‐2	97.80, SD = 10.57	101.82 SD = 9.87	99.80 SD = 11.49	102.12 SD = 11.66	–0.16	99.82 SD = 12.60	100.83 SD = 12.06	–0.27	100.10 SD = 11.60	103.45 SD = 10.85	–0.06
TOWK—Expressive Vocabulary	17.82 SD = 3.05	17.95 SD = 2.96	17.87 SD = 2.85	18.57 SD = 3.10	0.19	17.69 SD = 2.90	18.06 SD = 2.86	0.08	18.66 SD = 3.28	19.10 SD = 3.37	0.10
TOWK—Receptive Vocabulary	25.46 SD = 4.80	27.13 SD = 5.18	26.42 SD = 4.35	27.32 SD = 5.77	–0.15	26.16 SD = 5.62	27.40 SD = 4.82	–0.08	26.38 SD = 5.42	27.27 SD = 4.72	–0.15
TOWK—Multiple Contexts	8.89 SD = 4.85	10.14 SD = 4.78	9.51 SD = 4.85	11.64 SD = 5.10	0.18	8.56 SD = 4.41	9.79 SD = 4.43	–0.00	9.72 SD = 5.18	10.98 SD = 4.66	0.00
TOWK—Figurative Usage	17.95 SD = 47.48	21.10 SD = 7.18	20.30 SD = 6.72	23.14 SD = 7.40	–0.04	18.46 SD = 7.90	20.61 SD = 8.30	–0.13	20.74 SD = 7.57	22.74 SD = 7.39	–0.16

Notes: BPVS‐2 = British Picture Vocabulary Scale, 2nd edn; TOWK = Test of Word Knowledge.

*d* is calculated as the raw difference in progress between groups divided by the pooled standard deviation on the same measure at pre‐test (Morris 2008).

^*^ *p* < 0.05.

**Table 3:** Descriptive statistics by intervention group (raw scores) for intervention‐specific measures of narrative and vocabulary

	Control		Vocabulary				Narrative				Combined			
	Pre	Post	Pre	Post	*d* (compared with control)	*Z*, *p*	Pre	Post	*d* (compared with control)	*Z*, *p*	Pre	Post	*d* (compared with control)	*Z*, *p*
Story generation task	16.52 SD = 3.33	16.01 SD = 3.18	15.28 SD = 2.92	16.15 SD = 2.81	0.22^a^	*z* = 1.43, *p* = .154	15.29 SD = 2.66	18.44 SD = 4.26	0.95^c^	*z* = 5.87, *p* < .001	15.82 SD = 3.43	17.47 SD = 3.94	0.54^b^	*z* = 3.79, *p* < .001
Narrative checklist	39.68 SD = 10.17	41.97 SD = 9.72	39.60 SD = 11.04	44.18 SD = 10.03	0.23^a^	z = 0.34, p = .737	39.95 SD = 11.02	47.68 SD = 11.62	1.50^c^	*z* = 2.15, *p* = .031	39.57 SD = 10.11	46.35 SD = 12.75	0.44^a^	*z* = 0.60, *p* = .549
Vocabulary definitions	6.45 SD = 3.34	7.52 SD = 3.64	6.99 SD = 3.68	5.03 SD = 5.03	0.37^a^	*z* = 2.99, *p* = .003	6.36 SD = 3.22	8.18 SD = 3.50	0.24^a^	*z* = 1.85, *p* = .064	6.61 SD = 3.59	9.18 SD = 3.65	0.46^a^	*z* = 3.71, *p* < .001
Vocabulary idiom awareness	3.89 SD = 2.39	4.57 SD = 2.56	4.09 SD = 2.38	3.13 SD = 3.13	0.61^b^	*z* = 3.47, *p* = .001	4.03 SD = 2.25	4.94 SD = 2.61	0.09^a^	*z* = 0.61, *p* = .539	4.56 SD = 2.46	5.94 SD = 3.07	0.36^a^	*z* = 2.64, *p* < .008
Expressive vocabulary task	6.51 SD = 1.96	6.78 SD = 2.06	6.55 SD = 2.02	2.26 SD = 2.26	0.27^a^	*z* = 2.45, *p* = .014	6.52 SD = 1.72	6.94 SD = 1.79	0.09^a^	*z* = 0.73, *p* = .463	6.69 SD = 2.00	7.62 SD = 2.11	0.34^a^	*z* = 3.09, *p* < .002
Receptive vocabulary task	29.42 SD = 4.10	30.72 SD = 4.29	29.67 SD = 4.55	31.90 SD = 4.31	0.27^a^	*z* = 2.59, *p* = .010	29.03 SD = 5.53	30.70 SD = 4.25	0.07^a^	*z* = 0.74, *p* = .460	29.66 SD = 4.43	32.27 SD = 3.71	0.34^a^	*z* = 3.26, *p* = .001

Notes: *d* is calculated as the raw difference in progress between groups divided by the pooled standard deviation (SD) on the same measure at pre‐test (Morris 2008).

a *d* = 0.2 is a small effect;

b *d* = 0.5 is a medium effect; and

c *d* = 0.8 is a large effect.

Cohen's *d* was calculated as the raw difference in progress between groups divided by the pooled SD on the same measure at pre‐test (Morris 2008). It is clear that the three intervention groups showed small improvements on most measures compared with the waiting controls. Values of *d* ranged from –0.26 to 1.08 for the different intervention groups compared with the waiting control group.

Comparisons were also made between the combined narrative and vocabulary group and the two single intervention (vocabulary or narrative alone) groups. Perhaps unsurprisingly, the combined narrative and vocabulary group performed significantly better than the narrative‐only group on three measures of vocabulary (receptive vocabulary, expressive vocabulary and vocabulary idioms), in addition the combined group was significantly better than the vocabulary group, but significantly worse than the narrative only group on the story generation task (though all effect sizes were relatively small, Cohen's *d* = –.26 to .16).

All analyses were performed on an intention‐to‐treat basis. Analyses were conducted in Stata 15.1 (Stata Corp, College Station, TX, USA) and structural equation models (SEM) were estimated in Mplus 8.0 (Muthén and Muthén 1998–2017) with full information maximum likelihood estimators to allow for missing data and robust (Huber–White) standard errors to allow for the clustering of children within schools.

### Effects of interventions on standardized measures of narrative and vocabulary skills

The principal interest was to examine the extent to which the interventions produced improvements on two separable language factors: narrative and vocabulary skills. An initial confirmatory factor analysis on the pre‐test data showed that a two‐factor model with separable factors for narrative and vocabulary gave an excellent fit to the data (*χ* ^2^ (13) = 16.779, *p* = .21; root mean square error of approximation (RMSEA) = .028 [90% CI = 0.000–0.063; Comparative Fit Index (CFI) = .995; Tueker‐Lewis Index (TFI) = .992). In this model the narrative and vocabulary factors showed only a moderate correlation (*r* = .40), supporting the decision to treat these as separable aspects of language ability.

**Figure 2:**
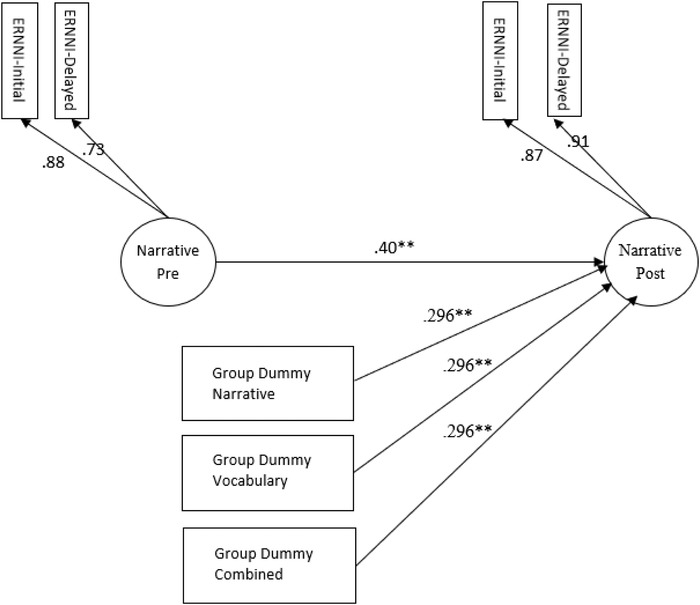
Effects of the interventions on narrative ability. Expression reception and recall of narrative instrument. The model shows the effects of the interventions on narrative skills at post‐test. Standardized coefficients are shown (except for dummy variables where *y*‐standardized values are shown). The *y*‐standardized dummy variable coefficients are equivalent to Cohen's *d*.

To assess the effects of the interventions on narrative ability we constructed the model shown in figure 2. In this model there is a narrative factor defined by the same two measures (ERRNI initial storytelling and ERRNI recall) at pre‐ and post‐test. This narrative factor captures the common variance shared by these two narrative measures. The model provides an excellent fit to the data (*χ* ^2^ (13) = 15.594, *p* = .27; RMSEA = .024 [90% CI = 0.000–0.060]; CFI = .995; TFI = .994). In this model the narrative latent variable shows weak factorial invariance across time (the unstandardized factor loadings were constrained to be equal). The narrative factor shows moderate longitudinal stability (*r* = .492). In this model the three unstandardized regression weights from each of the dummy codes (narrative versus control; vocabulary versus control; and combined versus control) were fixed to be equal. These constraints provide a direct test of whether the intervention effects differ in size from each other. Imposing these constraints resulted in a negligible change in model fit (*χ* ^2^ (2) = 0.023, *p* = .988) confirming that the size of the intervention effect did not differ between the three conditions. The most critical result from this analysis is that all three intervention groups show a significantly greater increase in their scores on the narrative post‐test factor (controlling for pre‐test scores) than the waiting control group (*d* = .296 [95% CI = 0.123–0.469]).

**Figure 3:**
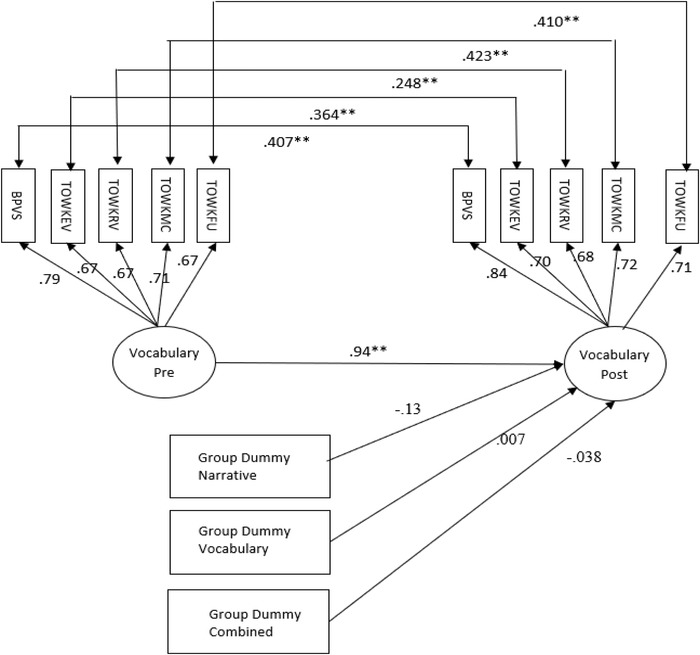
Effects of the interventions on vocabulary ability. BPVS = British Picture Vocabulary Scale, 2nd edn (BPVS‐2); TOWKEV = Test of Word Knowledge Expressive Vocabulary; TOWKRV = Test of Word Knowledge Receptive Vocabulary; TOWKMC = Test of Word Knowledge Multiple Contexts; TOWKFU = Test of Word Knowledge Figurative Usage. The model shows the effects of the interventions on vocabulary skills at post‐test. Standardized coefficients shown (except for dummy variables where *y*‐standardized values are shown). The *y*‐standardized dummy variable coefficients are equivalent to Cohen's *d*.

To assess the effects of the interventions on vocabulary knowledge we used the model shown in figure 3. In this model a vocabulary factor is defined by the same five standardized measures of vocabulary knowledge (the BPVS‐2, and the four subscales of the TOWK; receptive and expressive vocabulary, comprehension of words in multiple contexts, and figurative language use) at pre‐ and post‐test. This vocabulary factor captures the common variance shared by the five vocabulary measures. The model provides a good fit to the data (*χ* ^2^ (60) = 93.895, *p* = .27; RMSEA = .040 [90% CI = 0.023–0.055]; CFI = .982; TFI = .978). In this model the vocabulary latent variable shows weak factorial invariance across time (the unstandardized factor loadings were constrained to be equal) and high longitudinal stability (*r* = .938).

The most critical result from this analysis is that none of the intervention groups shows a significantly greater increase in its scores on the vocabulary post‐test factor (controlling for pre‐test scores) than the waiting control group, with all effect sizes being negligible (vocabulary group *d* = .007 [95% CI = –.235 to .248]; narrative group *d* = –.128 [95% CI = –0.312 to 0.056]; combined narrative and vocabulary group *d* = –.038 [95% CI = –0.213 to 0.136]).

### Effects of interventions on directly taught narrative and vocabulary skills

We also wanted to assess the effects of the interventions on measures of directly taught skills, including the narrative checklist and story generation task, as well as the vocabulary definitions, idiom awareness, expressive and receptive vocabulary.

We assessed differential effects of the three interventions on each of these measures in a series of mixed effects regression models (ANCOVA models) with post‐test scores as the outcome measure, the corresponding pre‐test score as a covariate, and group represented by three dummy codes contrasting each intervention group (vocabulary, narrative, combined narrative and vocabulary) with the waiting controls. In each model we used varying intercepts but fixed slopes across schools, since in no case did using varying slopes yield a significant improvement in the fit of the model.

For the narrative checklist, the narrative group showed significant improvements compared with the waiting control group (marginal mean group difference = 15.83 [95% CI = 1.43–30.23]; *z* = 2.15, *p* = .031, *d* = 1.50) but neither the vocabulary group (marginal mean group difference = 2.50 [95% CI = –12.08 to 17.08]; *z* = 0.34, *p* = .737, *d* = .23) nor the combined narrative and vocabulary group (marginal mean group difference = 4.40 [95% CI = –9.99 to 18.80]; *z* = 0.60, *p* = .549, *d* = .43) showed significant improvements.

For the story generation task, the narrative (marginal mean group difference = 2.92 [95% CI = 1.95–3.90]; *z* = 5.87, *p* < .001, *d* = .95) and combined narrative and vocabulary groups (1.85 [95% CI = –0.89 to 2.81]; *z* = 3.79, *p* < .001, *d* = .54) both showed significant improvements compared with the waiting control group, whereas the vocabulary group did not (marginal mean group difference = .71 [95% CI = 0.27–1.68]; *z* = 1.43, *p* = .154, *d* = .22).

Finally, for the four vocabulary measures (vocabulary definitions, idiom awareness and expressive and receptive vocabulary), the two groups that had received vocabulary intervention showed significant gains from intervention compared with the waiting control group whereas the narrative group did not. For the vocabulary group there were significant gains on vocabulary definitions (marginal mean group difference = 1.30 [95% CI = 0.45–2.15]; *z* = 2.99, *p* = .003, *d* = .37), idiom awareness (marginal mean group difference = 1.14 [95% CI = 0.50–1.79]; *z* = 3.47, *p* = .001, *d* = .61) expressive vocabulary (marginal mean group difference = .53 [95% CI = 0.11–0.96]; *z* = 2.45, *p* = .014, *d* = .27) and receptive vocabulary (marginal mean group difference = 1.16 [95% CI = 0.28–2.04]; *z* = 2.59, *p* = .010, *d* = .27). There were also significant improvement in the combined narrative and vocabulary group on these measures (vocabulary definitions, marginal mean group difference = 1.60 [95% CI = 0.76–2.45]; *z* = 3.71, *p* < .001, *d* = .46; idiom awareness marginal mean group difference = .87 [95% CI = 0.23–1.52]; *z* = 2.64, *p* < .008, *d* = .36; expressive vocabulary marginal mean group difference = .67 [95% CI = 0.24–1.09]; *z* = 3.09, *p* < .002, *d* = .34 and receptive vocabulary marginal mean group difference = 1.45 [95% CI = 0.58–2.32]; *z* = 3.26, *p* = .001, *d* = .34). In contrast the narrative only group did not show any significant improvement in comparison to the waiting control group on these four measures (vocabulary definitions marginal mean group difference = .80 [95% CI = –0.05 to 1.65]; *z* = 1.85, *p* = .064, *d* = .24) idiom awareness (marginal mean group difference = .20 [95% CI = –0.44 to 0.84]; *z* = 0.61, *p* = .539, *d* = .09) expressive vocabulary (marginal mean group difference = .16 [95% CI = –0.26 to 0.58]; *z* = 0.73, *p* = .463, *d* = .09) and receptive vocabulary (marginal mean group difference = .33 [95% CI = –0.54 to 1.20]; *z* = 0.74, *p* = .460, *d* = .07).

## Discussion

This paper has presented the results of an RCT evaluating the efficacy of three different forms of language intervention for students in secondary school (Year 7) with language difficulties. The interventions were delivered by TAs over 6 weeks and we compared a narrative, vocabulary and combined narrative and vocabulary intervention with an untreated (waiting control) control group. The results are clear in showing reliable improvements in all three intervention groups on a latent narrative skills factor (defined by measures from the ERRNI) with moderate effect sizes. In contrast there were no effects of the interventions on a latent vocabulary factor reflecting standardized measures of vocabulary knowledge.

The positive effects of all three interventions on narrative skills are encouraging especially in light of the fact that the intervention was of short duration (three small group sessions each week over 6 weeks). The positive effects of the interventions here align with results from studies with much younger children (e.g., Davies *et al*. 2004, Fricke *et al*. 2013) and with findings from smaller scale studies with adolescents (e.g., Gillam *et al*. 1995, Joffe 2006, Stringer 2006). Although the three interventions differed in their emphasis, all three programmes involved getting students to produce language in a structured and supportive environment with feedback. It can be argued that the changes in narrative skills observed here may reflect ‘generative’ language skills; applying common strategies that generalize to different language contexts.

In contrast to the results for narrative, none of the interventions here produced reliable improvements on standardized measures of vocabulary knowledge. The absence of improvements in vocabulary knowledge is not surprising given the short duration of the intervention. Vocabulary knowledge is to a large extent item specific, so finding generalized improvements on standardized tests is going to be difficult, as is reflected by the mixed results reported in other studies with the same age group (e.g., Starling *et al*. 2012, Murphy *et al*. 2017). Although one study with younger children (e.g., Clarke *et al*. 2010) did report effects of language intervention on standardized measures of untaught vocabulary, this study was of much longer duration (3 × 30‐min sessions for 20 weeks). Speculatively, it seems likely that such effects reflect quite general changes in children's metacognitive strategies and more active engagement in language learning which will take significant amounts of time to take effect.

For measures of directly taught skills we did find differential effects between the narrative and vocabulary interventions, with the narrative intervention group and combined narrative and vocabulary intervention group performing significantly better than the waiting control group on the narrative checklist and both the narrative and combined narrative and vocabulary intervention group performing better on the story generation task. Hence, there were some improvements noted in both implicit story telling skills (story generation task) and explicit narrative knowledge (narrative checklist) as a result of the intervention. This was not the case with the vocabulary intervention group. Similarly, the vocabulary intervention group and the combined narrative and vocabulary intervention group performed significantly better than the waiting control group on all the intervention‐specific vocabulary measures including receptive and expressive vocabulary, vocabulary definitions and idiom awareness. This pattern was again, not evident with the narrative intervention group. The combined narrative and vocabulary intervention group, receiving both narrative and vocabulary intervention, within the same time period, made significant improvements on one narrative measure (story generation) and on all vocabulary tasks compared with the waiting control group. Interestingly, the combined narrative and vocabulary group show progress in both types of outcome, despite having less focused time on each component, suggesting the possibility of an enhanced cumulative effect when combining narrative and vocabulary elements.

Whilst significant intervention effects were obtained from intervention‐specific measures for both narrative and vocabulary skills, this was not replicated with standardized assessments. This is consistent with Marulis and Neumann's (2010) assertion that standardized tests are not always sensitive enough to detect changes during a time‐limited period of intervention. They advise the combined use of standardized and non‐standardized measures to monitor change, as was adopted in this study.

One important distinction from previous research (e.g., Lowe and Joffe 2017, Spencer *et al*. 2017b, Wright *et al*. 2018), which focuses on targeting the stimuli (e.g., specific idioms or word definitions), is that this study noted improvements across a range of stimuli that were not necessarily targeted in the intervention. This suggests that some generalized learning may have taken place beyond the specific stimuli targeted in the interventions.

### Strengths and limitations

This RCT provides robust evidence for a causal effect on narrative skills from three related language interventions delivered over a relatively short time period in secondary school settings. The size of the effects here were modest, but that is not surprising given the short duration of the intervention. The vocabulary outcomes were more mixed, with positive findings only evident for the intervention‐specific measures. Our findings have clear educational implications in showing that targeted language intervention work can be effective for secondary school students. The interventions are educationally realistic and make use of existing school resources (TAs). This type of service delivery model has important pedagogic benefits in that it provides targeted intervention in the school setting, enhances the knowledge and skills of TAs working with adolescents with language difficulties, and has potential economic and resource advantages.

The response rate from the schools was good and the attrition was low. The study design, with each TA giving all three interventions, effectively controlled for any potential differences in quality between TAs (e.g., individual differences in ability, experience etc.). However, because the TAs delivered all the interventions, it may have sometimes been difficult to compartmentalize and keep the narrative and vocabulary interventions separate. Another limitation of the current study was the limited duration of the intervention. Future studies are needed that adopt the approach used here but implement it over much longer periods (e.g., over a whole school year). The students targeted here have, by definition, shown poor rates of language development across an extended period of development, and it is likely that they will need intervention over an extended period of time to ameliorate their language weaknesses.

## Summary and conclusions

Adolescents with weak language skills made significantly greater improvements on standardized measures of narrative, but not vocabulary skills compared with controls. Intervention‐specific measures, albeit less reliable, showed differential intervention effects for both narrative and vocabulary. The results show that educationally realistic language interventions can be delivered in secondary schools at relatively low cost. Future studies are required to explore the efficacy of much longer duration language interventions for the population studied here. Such interventions have the potential to deliver important educational benefits in a cost effective way.
